# Fabrication of AChE/SnO_2_-cMWCNTs/Cu Nanocomposite-Based Sensor Electrode for Detection of Methyl Parathion in Water

**DOI:** 10.1155/2018/2874059

**Published:** 2018-06-06

**Authors:** Vikas Dhull

**Affiliations:** Department of Biotechnology Engineering, University Institute of Engineering & Technology, Maharshi Dayanand University, Rohtak 124001, Haryana, India

## Abstract

The work highlights inhibition-based* Acetylcholinesterase* (AChE) fabrication using composite nanomaterial comprising tin oxide nanoparticles (SnO_2_) and carboxylated multiwalled carbon nanotubes (cMWCNTs) for detection of pesticide methyl parathion (MP) in water samples. Working electrode AChE/SnO_2_-cMWCNTs/Cu exhibited high sensitivity with a linearity range of 1.0 *μ*M to 160 *μ*M and a minimum detection limit of 0.1 *μ*M for MP in water. The fabricated electrode was found biocompatible and nontoxic which can be used to detect low concentrations of pesticide in water samples. The synergistic and facilitated electron transferring properties of SnO_2_-cMWCNTs/Cu made it an excellent support for immobilization of enzyme in sensing technology. The enzyme AChE was covalently immobilized with cMWCNTs using glutaraldehyde as crosslinking agent which has enhanced the storage stability and reusability of the method. The reusability attained was 30 times for 40 days when AChE/SnO_2_-cMWCNTs/Cu was stored at low temperature of 4°C. Developed sensor showed excellent analytical recovery of pesticide in water sample with negligible effect of interfering species. Also, AChE/SnO_2_-cMWCNTs/Cu was easily reactivated simply by varying pH of phosphate buffer. This method is fast, reliable, and accurate showing successful development of amperometric biosensor for detection of MP in water sample.

## 1. Introduction

In the present scenario, metal oxides are playing a dynamic role in research field. The properties of metal oxides make them an important candidate for various analytical fields. Their possessions are highly dependent on size. The metal oxides possess excellent electrocatalytic properties and this enhancement in electron transfer is achieved due to decreasing size. Consequently, these metal oxides have been used for nanomaterial synthesis as zinc oxide nanoparticles, gold nanoparticles, silver nanoparticles, and so forth. They have found wide range of application in analytical fields for development of sensors, as antimicrobial activity, catalysis, fabrication of nanomaterial-based electrodes, for surface coating, photovoltaic devices, dye removal from effluents, and many more.

Among the abovementioned metal oxides tin oxide (SnO_2_) has been less exploited. SnO_2_ being an important material possesses a broad visible spectrum, high thermal stability, and excellent physiochemical properties [1]. SnO_2_ is highly stable as it is n-type semiconductor having band gap of 3.6 eV [2]. For the above property the SnO_2_ nanoparticles can be used as a promising material for fabrication of optoelectronic devices [3], solar cells [4], lithium ion batteries [5], and sensors and transistors for detection of combustible gases [6–9]. There are many methods available for synthesis of SnO_2_ nanomaterials which include sonochemical method [10], solvothermal [11], sol gel [12], hydrothermal [13], microwave technique [14], and coprecipitation method [15].

The nanoparticles have been successfully used for fabrication of biosensors for detection of various analytes. The pesticides have been extensively used for enhanced crop productivity, but on the other hand it is also causing serious health concerns in humans which can cause irritation to skin, allergy, and accumulation of neurotransmitters, as well as affecting central nervous system finally resulting in death [16]. Different supports have been used for biosensor fabrication [17]. Nanomaterial-based supports for detection of pesticides in various samples include carbon nanotube based working electrode [18], zinc oxide nanoparticles with multiwalled carbon nanotubes (MWCNTs) [19], gold (Au)-platinum (Pt) bimetallic nanoparticles (NPs)/glassy carbon electrode (GCE) [20], MWCNTs-Au nanocomposites/GCE [21], AuNPs-MWCNTs/GCE [22], AuNPs/GCE [23], and zirconium dioxide/chitosan/GCE [24]. Among the nanoparticles SnO_2_ nanoparticles have been less exploited for pesticide detection. So, this research is focused on SnO_2_ nanoparticles chemical synthesis using coprecipitation method. It is easy to synthesize SnO_2_ nanoparticles using chemical synthesis, it is simple, fast, and reliable and pure nanoparticles have been obtained. Further, synthesized nanoparticles have been used to fabricate a working electrode AChE/SnO2-cMWCNTs/Cu for pesticide detection in water samples.

## 2. Experimental

### 2.1. Chemicals and Instrumentation

Sodium carbonate anhydrous, Triton X-100, and glutaraldehyde were from Merck Limited, Mumbai, India. Stannic chloride anhydrous, methanol and ethyl alcohol, and carboxylated multiwalled carbon nanotubes were from Sisco Research Laboratories Pvt. Ltd., Mumbai, India. Distilled water was used throughout the experiment where needed. Pesticide methyl parathion (MP) and paraffin oil were purchased from local market. All the chemicals used throughout experiment were of analytical grade. Shimadzu UV-Vis Spectrophotometer (UV 2450) was used for absorbance measurement, and Fourier transform infrared (FTIR) spectroscopy was performed in Department of Biotechnology Engineering, University Institute of Engineering & Technology, Maharshi Dayanand University, Rohtak. Particle size analyzer (PSA) was performed using Malvern instrument at Guru Jambheshwar University of Science and Technology, Hisar. Transmission Electron Microscopy (TEM) was done using microscope Model JEM-2100F and Scanning Electron Microscopy (SEM) at Advanced Instrumentation & Research Facility (AIRF), Jawaharlal Nehru University (JNU), New Delhi. Electrochemical analysis was done using Potentiostat Model 910 PSTAT mini at Centre for Biotechnology, Maharshi Dayanand University, Rohtak. HPLC instrument was from YL Instruments, Korea.

### 2.2. Synthesis of Tin Oxide Nanoparticles

For synthesizing SnO_2_ nanoparticles hydrated stannic chloride is mixed with distilled water. The above solution was taken in a 500 ml beaker and put on the magnetic stirrer for continuous stirring using a magnetic bead. The solution was kept on constant stirring for 10 minutes to achieve a homogeneous solution of stannic chloride. The stock solution of anhydrous sodium carbonate was also prepared and used to add in a drop wise manner to the stirring stannic chloride solution. After 1-hour color of stannic chloride solution turns white which may be due to precipitate formation after addition of anhydrous sodium carbonate. The solution having white precipitates was kept on continuous stirring for 30 minutes. Then, solution was left undisturbed for 3 hours to allow precipitates to settle down. The precipitates were collected by decanting extra solution and dried using filter paper. The impurities of obtained precipitates were removed by washing with ethyl alcohol. The above procedure was repeated for 5 times in order to get suitable weight of precipitates. Obtained precipitates were collected and crushed properly using a pestle mortar. Finally, obtained particles were treated as pure SnO_2_ nanoparticles and used further in experiment.

### 2.3. Characterization of Tin Oxide Nanoparticles

Tin oxide nanoparticles were characterized using UV-Visible spectroscopy for testing the absorbance of nanoparticles. This is the initial confirmation of successful confirmation of SnO_2_ nanoparticles. The above synthesized nanoparticles were subjected to FTIR to analyze bonds present in nanoparticles. PSA was done for determining average size of nanoparticles. Further, characterization includes nanoparticle analysis using TEM. This confirmed shape and size of SnO_2_ nanoparticles synthesized in laboratory.

### 2.4. Fabrication of Sensor Electrode

**Figure 1:**
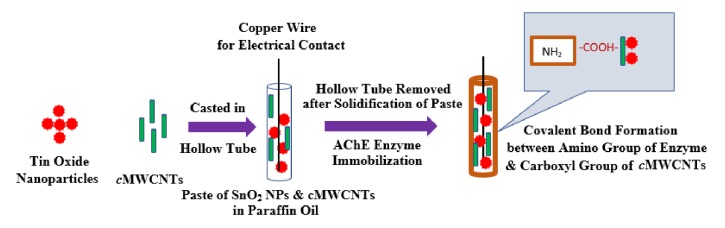
Fabrication strategy of working electrode of developed sensor.

The fabrication of sensor electrode consists of forming a composite paste of SnO_2_ nanoparticles and cMWCNTs in paraffin oil. Then, paste was filled in a plastic hollow tube (1 mm × 2 cm). A fine copper wire was inserted in tube for electrical contact. The plastic tube having paste was left for drying overnight at room temperature in order to dry the paste. After drying, outer covering of tube was cut and resulting material was treated as core body of working electrode. The core electrode was then dipped in the solution of enzyme acetylcholinesterase which was prepared in glutaraldehyde. This will allow uniform coating of enzyme along with crosslinker glutaraldehyde on core of working sensor electrode. A covalent bond will be formed due to presence of carboxyl group on MWCNTs and amino group present on enzyme. The fabricated sensor electrode was stored at 4°C when not in use. The fabrication strategy of working electrode of sensor was represented in Figure 1.

### 2.5. Electrochemical Analysis

A three-electrode system was used for performing electrochemical analysis. The above fabricated working electrode along with Auxiliary Electrode (Pt) and Reference Electrode (Ag/AgCl) constituted assembly of sensor. Electrochemical assembly also included reaction mixture which is comprised of substrate (acetylthiocholine chloride), testing buffer (phosphate buffer). Before use working electrode was calibrated at +0.4 V to achieve steady state current using the phosphate buffer having pH 7.5. After successful calibration cyclic voltammetry study was investigated from +0.0 to +1.0 V. As, this is inhibition-based sensor so current generated is inversely proportional to amount of pesticide present in the sample.

### 2.6. Evaluating the Newly Fabricated Sensor

The newly fabricated electrode was evaluated on a variety of parameters including linear working range, minimum limit of detection, pesticide analytical recovery, and precision and accuracy of developed sensor. The sensor was also evaluated for storage stability and reusability. Linearity and minimum detection limit was calculated by correlating with standard plotted graph. For investigating reliability of developed method different concentrations of pesticide (methyl parathion) were added to sample and mean of analytical recoveries was recorded. The sensor was exploited for analyte recoveries within batch and between batches for determining reliability of method. Accuracy was checked by testing water samples spiked with different pesticide concentrations using standard method HPLC (*x*) and newly developed sensor (*y*). Storage stability of the sensor was investigated after storing newly fabricated working electrode for 2 months. The pesticide concentration recovered was analyzed on every alternate day for storage stability. The pesticide was also determined in the water samples collected from Pond near agricultural field, agriculture runoff water, and waste water near villages of Rohtak, Haryana. Reverse Phase HPLC analysis was conducted using C-18 column, UV-Vis detector at 225 nm, and 20 *μ*L of sample was injected manually. The isocratic mobile phase was developed using water and methanol (65:35 v/v) at pH 5.3. The flow rate was adjusted at 1.2 ml/min.

## 3. Results and Discussion

### 3.1. Characterization of Tin Oxide Nanoparticles

**Figure 2:**
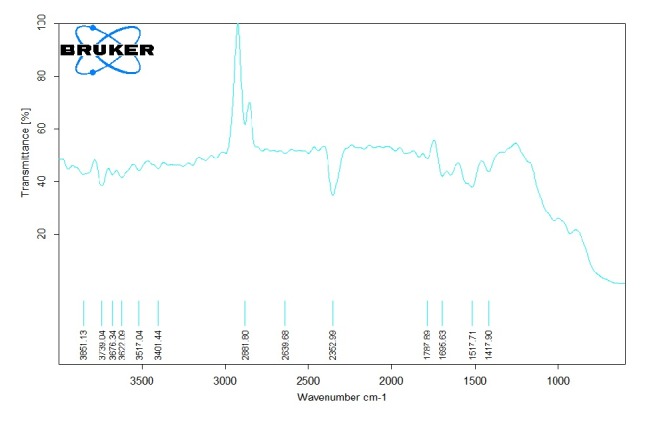
Peaks of lab synthesized SnO_2_ nanoparticles using Fourier transform infrared spectroscopy.

**Figure 3:**
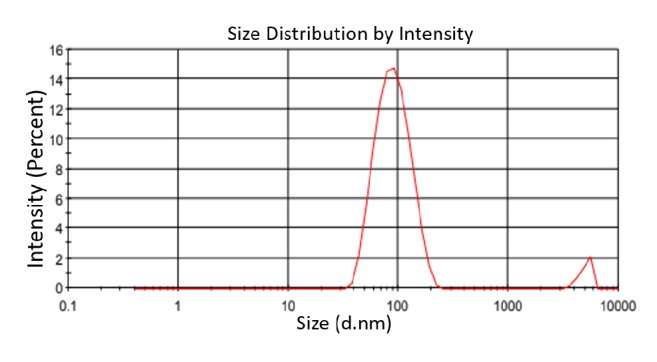
Particle size analysis of nanoparticles.

**Figure 4:**
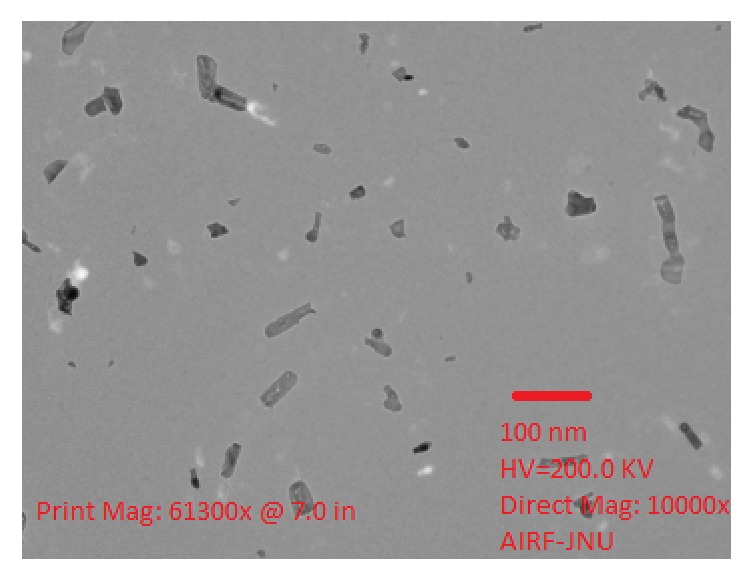
Transmission Electron Microscopic Image of synthesized SnO_2_ nanoparticles shown on 100 nm scale with direct magnification of 10000x.

**Figure 5:**
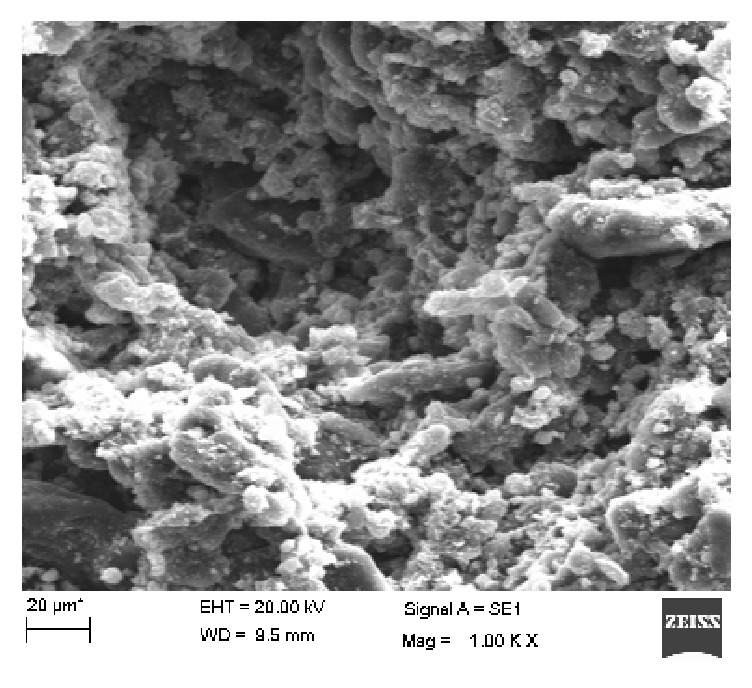
Representing Scanning Electron Microscopic image of AChE/SnO_2_-cMWCNTs/Cu.

The UV-Vis spectrum of synthesized nanoparticles was recorded at 350 nm which proves the successful synthesis of SnO_2_ nanomaterial. The nanoparticles were then subjected to FTIR analysis which also showed the absorption spectra of SnO_2_ nanoparticles. The absorbance peaks were recorded at 1695.63 cm^−1^ to 1787.89 cm^−1^ which corresponds to C=O stretch, 2639.68 cm^−1^ to 2881.80 cm^−1^ corresponding to O-H stretch, and 3739.04 cm^−1^ to 3851.13 cm^−1^ representing N-H stretch as shown in Figure 2. These bending vibrations are signature peaks of SnO_2_ nanoparticles and are responsible for formation of covalent bonding between enzyme and support. The average size of the synthesized nanoparticles was also determined using PSA which showed that synthesized particles were in nanorange as shown in Figure 3. Further, Transmission Electron Microscopy (TEM) was performed which finally confirmed nanosized synthesis of SnO_2_ nanoparticles and their rod-like shape Figure 4. The rod-like structures of nanoparticles are also helpful in enhanced electrocatalytic activity and excellent for sensing purpose. These nanoparticles were mixed with cMWCNTs in paraffin oil and enzyme acetylcholine esterase was immobilized on it. This was treated as working electrode of sensor and characterized by SEM as shown in Figure 5.

### 3.2. Electrochemical Analysis Using Sensor Electrode

**Figure 6:**
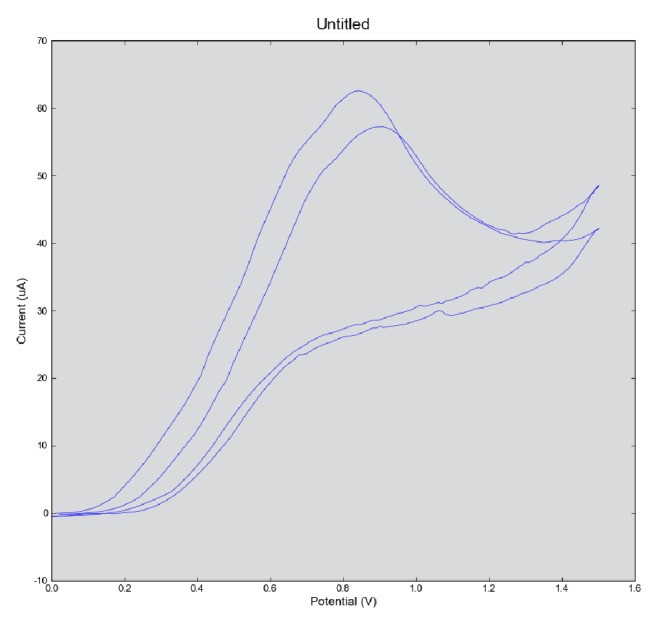
Cyclic voltammetry study of sensor electrode for electrochemical analysis.

The working electrode AChE/SnO_2_-cMWCNTs/Cu electrode was used for recording cyclic voltammetry. Analysis was done from +0.0 to +1.6 V at two scan rates of 25 mV/s and 50 mV/s using phosphate buffer of pH 7.5 and temperature was 35°C. The highest peak was recorded at +0.85 V with a scan rate of 50 mV/s as shown in Figure 6. This peak is due to breakdown of substrate ATCI by enzyme AChE immobilized on sensor electrode and treated as optimum working potential for further experiment. This potential was equal to carbon nanotube electrode (0.85 V) [29] and carbon paste electrode (0.85 V) [30, 31] and lower than mesoporous carbon and carbon black electrode (0.9 V) [32] and screen printed carbon electrode (0.9 V) [33] for pesticide detection using organophosphorus hydrolase enzyme. The decline in optimal working potential of AChE/SnO_2_-cMWCNTs/Cu was maybe due to increased surface area of rod-like SnO_2_ nanoparticles and higher electrocatalytic activity.

### 3.3. Evaluation of Fabricated Sensor Electrode

**Figure 7:**
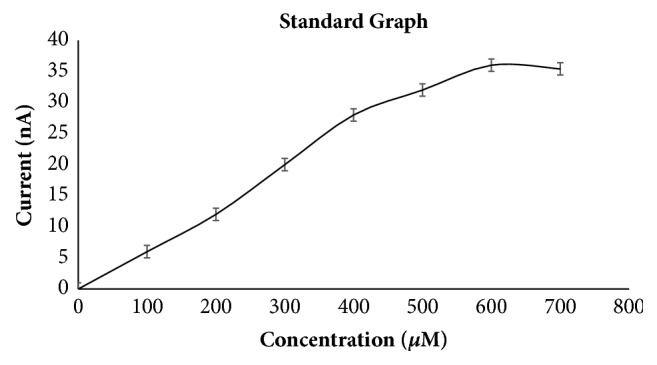
Standard graph between response current and substrate concentration.

For evaluating developed electrode, a standard graph was plotted between substrate concentration and response current as shown in Figure 7. This curve was then used to determine linearity of electrode and minimum limit of detection. The sensor response was found to be linear from 1 *μ*M to 160 *μ*M. The linearity of newly fabricated sensor is better than earlier reported methods using variety of other nanomaterials for pesticide detection carbon paste electrode (4.6 to 46 *μ*M, methyl parathion and paraoxon) [34], silver nanocubes synthesis for determining paraoxon [35], pore glass beads (up to 140 *μ*M for methyl parathion and up to 120 *μ*M for paraoxon) [36], and gold electrode (1–10 *μ*M for methyl parathion and paraoxon) [37]. The minimum concentration of pesticide the sensor can detect is 0.1 *μ*M. This minimum detection limit is better than earlier reported methods using MWCNT/GCE (0.314 *μ*M of paraoxon) [38] and modified GCE (150 nM of paraoxon ethyl) [20]. The developed sensor possesses good linearity and detection limit so this is better and sensitive compared to some earlier reported methods.

**Figure 8:**
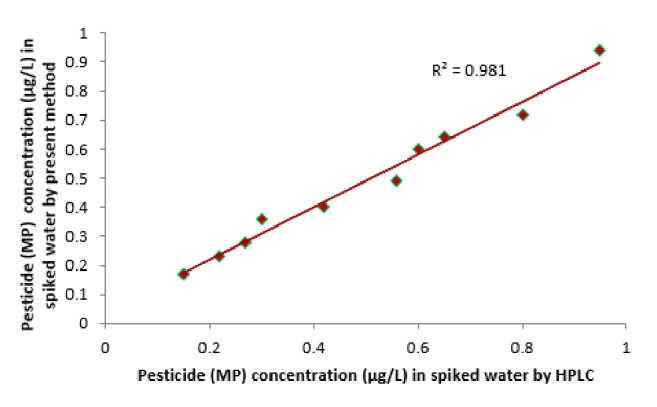
Correlation of standard method HPLC (*x*) with the newly developed method (*y*).

**Figure 9:**
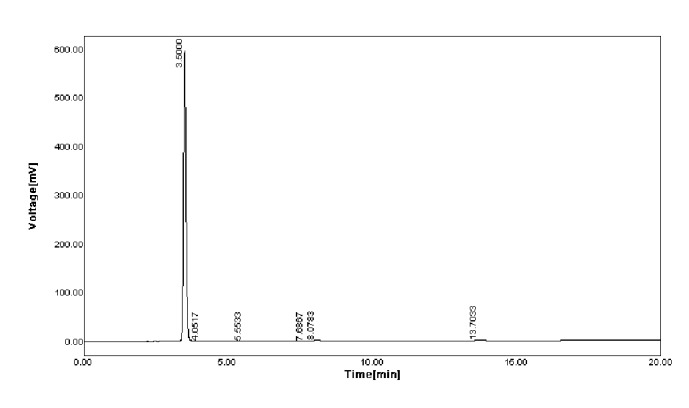
HPLC chromatogram of Standard Pesticide solution.

**Figure 10:**
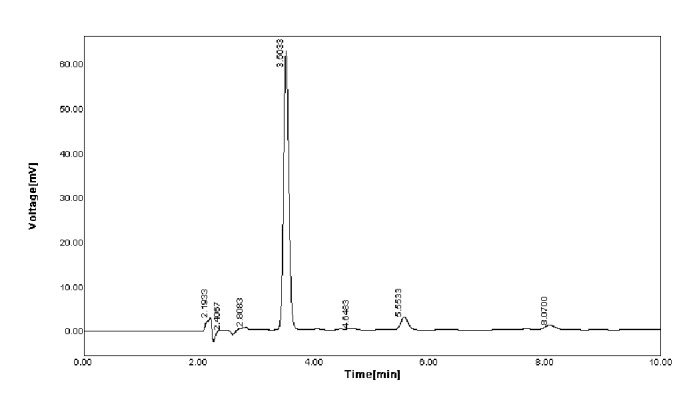
HPLC chromatogram of spiked water sample.

Reproducibility of the present method was checked by spiking water samples with known concentration (10 *μ*M, 15 *μ*M, 20 *μ*M, and 25 *μ*M) of pesticide (MP) as shown in Table 1. The amount of pesticide recovered is good enough. The reproducibility was also investigated at different time intervals on the same day (within batch) with good coefficient of variance (CV) > 1 and after storage for long time (between batches) with CV > 1. The present method is also correlated with standard HPLC method as shown in Figure 8. A good correlation has been achieved with a *R*^2^ value of 0.981. This proved developed method is reliable and accurate. The pesticides determined in different collected water samples were also analyzed by the fabricated sensor as shown in Table 2. Including this HPLC analysis was done for Standard Pesticide solution (100 *µ*M) as shown in Figure 9 and spiked water sample (20 *µ*M) as shown in Figure 10. It was revealed that retention time of pesticide (methyl parathion) was at 3.5 min which was also correlated with spiked sample.

### 3.4. Reusability and Storage Stability

The developed sensor was stored at 4°C for 60 days and its analytical activity was analyzed. Fabricated sensor was stable up to 40 days and after this a decline has been recorded in efficiency of sensor for peptide detection. This is better than earlier reported methods for pesticide detection using other nanomaterials [39, 40]. The sensor electrode was washed after every use with phosphate buffer of pH 8.0 in order to reactivate the catalytic sites of enzymes occupied by substrate. The varying pH made conformational changes on the active site of enzyme and releases the attached substrate leading to reactivation of enzyme.

### 3.5. Conclusion

The research is focused on characterization of synthesized SnO_2_ nanoparticles-based sensing electrode. The sensor electrode consisted of composite of SnO_2_ nanoparticle and cMWCNTs and the AChE enzyme. Characterization techniques include UV-Vis spectroscopy for absorbance, Fourier transform infrared spectroscopy for bending pattern, Transmission Electron Microscopy for shape and size of nanomaterial, and Scanning Electron Microscopy to study surface morphology. These techniques successfully confirmed fabrication of electrode. Electrochemical analysis of sensor electrode showed low working potential +0.85 V at scan rate of 50 mV/s which is better than earlier reported methods. This may be due to rod-like shape of SnO_2_ which has increased surface area along with cMWCNTs. This combination may have enhanced the electrocatalytic activity of sensor. The sensor was linear from range of 1 *μ*M to 160 *μ*M which is better performance with lower detection limit of 0.1 *μ*M for pesticide determination in water samples. This sensor electrode was reusable for 30 times and reproduced successfully. This electrode can be stored stably for 40 days at 4°C. This may be due to less leaching of enzyme from the electrode as a result of covalent bond formed between enzyme and carboxyl group present on cMWCNTs present in core body of electrode along with nanoparticles. The sensor, fabricated in this work, is better amperometric method for pesticide determination in water samples. The present method is compared with the earlier reported methods as shown in Table 3.

## Figures and Tables

**Table 1:** Representing percent analytical recovery of Pesticide (MP) added and recovered.

Pesticide (MP) added (*μ*M)	Pesticide (MP) recovered (*μ*M) with mean of (*n* = 5)	Pesticide (MP) % Recovery	Standard Deviation
10	9.25	99.0	0.1
15	14.21	98.2	0.09
20	19.34	99.1	0.07
25	24.47	99.2	0.1

**Table 2:** Analysis of water samples using the newly fabricated sensor.

Sr. No.	Sample	Current (nA)	Concentration (*μ*M)
1.	Standard Sample	6	100
2.	Pond Near Agriculture Field	0.5	8.3
3.	Agriculture runoff water	11	183
4.	Waste Water	0.2	3.3

**Table 3:** Comparison of present method with earlier reported methods.

Detection Method	Electrode Material	Immobilization Method	Detection Limit	Linearity	Compound Detected	Storage Stability	Reference
Amperometric	SiSG & AuNPs	Adsorption	0.6 ng/ml	2-15 *μ*g/ml	Monocrotophos	30 Days	[25]
Amperometric	AuNPs/Au electrode	Adsorption	33 × 10^−3^ *μ*M	10 × 10^−3^-135 × 10^−3^ *μ*M	Carbofuran	7 Days	[26]
Amperometric	PAN/AuNPs/Pt electrode	Covalent	0.026 × 10^−5^ *μ*M	3.6 × 10^−7^-3.6 × 10^−4^ *μ*M	Paraoxon	30 Days	[27]
Amperometric	CHIT and GNPs/Au	Chemisorption	0.1 × 10^−3^ *μ*M	0.3 × 10^−3^-60.5 × 10^−3^ *μ*M	Malathion	NR	[28]
Amperometric	SnO2-cMWCNTs/Cu	Covalent	0.1 *μ*M	1.0 *μ*M to 160 *μ*M	Methyl Parathion	40 Days	Present Method

## Data Availability

All the necessary data related to manuscript is included in manuscript.
